# Effectiveness of the Information–Motivation–Behavioral Skills Model-Based Nursing Intervention on Maternal and Neonatal Outcomes in Women with Gestational Diabetes Mellitus: A Randomized Controlled Trial

**DOI:** 10.1177/26884844251380133

**Published:** 2025-09-26

**Authors:** Caihua Shao, Shunlian Ma, Xia Liu

**Affiliations:** ^1^ Department of Obstetrics, Aksu First People’s Hospital, Aksu, China.; ^2^ Cadre Health Care Department, Aksu First People’s Hospital, Xinjiang, Aksu City, Aksu, China.

**Keywords:** gestational diabetes mellitus, nursing intervention, information–motivation–behavioral skills model, maternal and infant outcomes, postpartum glucose metabolism

## Abstract

**Objective:**

This study aimed to evaluate the effectiveness of the information–motivation–behavioral skills (IMB) model-based nursing intervention in improving maternal and neonatal outcomes in patients with gestational diabetes mellitus (GDM).

**Methods:**

A total of 240 pregnant women with GDM were enrolled and randomly assigned to either the IMB group or the traditional health education group between June 2023 and May 2024. The IMB group received a 6-week intervention incorporating information support, motivational enhancement, and behavioral skill development. The control group received standard health education. Outcomes assessed included fasting plasma glucose (FPG), glycosylated hemoglobin (HbA1c), insulin treatment rates, adverse delivery outcomes, and postpartum glucose metabolism abnormalities.

**Results:**

The IMB group showed a significant reduction in FPG from 8.05 mmol/L (145.05 mg/dL) to 4.95 mmol/L (89.19 mg/dL) (*p* < 0.001) and HbA1c from 6.60% to 4.98% (*p* < 0.001) postintervention, compared with the control group’s reduction to 6.00 mmol/L (108.11 mg/dL) (*p* < 0.001) and 5.78% (*p* < 0.001), respectively. The rate of insulin treatment was 2.5% in the IMB group versus 13.3% in the control group (*p* = 0.003). The IMB group had a lower cesarean section rate (15.0% vs. 40.0%, *p* < 0.001) and neonatal respiratory distress syndrome incidence (1.67% vs. 10.0%, *p* = 0.007). Postpartum, the IMB group exhibited a lower total incidence of abnormal glucose metabolism (12.5%) compared to the control group (25.0%, *p* < 0.001).

**Conclusion:**

The IMB model-based nursing intervention was more effective in managing blood glucose levels, reducing the need for insulin therapy, and improving both maternal and neonatal outcomes compared to traditional health education. This intervention may offer a promising approach to enhance the care of women with GDM.

## Introduction

Gestational diabetes mellitus (GDM), characterized by glucose intolerance of varying degrees that first occurs during pregnancy, stands as one of the most prevalent complications in this period.^1^ The global incidence of GDM ranges from 1% to 25%, influenced by differing diagnostic criteria and racial disparities.^2^ According to some research reports, the incidence rate of GDM in China reached 14.8%, and with the country’s fertility policy adjustments and the increasing prevalence of advanced maternal age and obesity, this figure continues to rise.^3,4^ GDM poses significant threats and challenges to both maternal and infant health, with risks including miscarriage, preterm birth, polyhydramnios, cesarean section, and a 50%–73% chance of recurrence in subsequent pregnancies.^5–7^ Women with a history of GDM are 10 times more likely to develop type 2 diabetes within 5–10 years postpartum, and their risk of cardiovascular diseases is also heightened compared to the general female population.^8^ Offspring of mothers with GDM are at risk of macrosomia, neonatal hypoglycemia, and pathological jaundice, with an increased likelihood of becoming overweight or obese in adulthood, potentially leading to type 2 diabetes.^9^ Once a woman is diagnosed with GDM, effective management becomes crucial.

Dietary and exercise interventions are important adjuvant therapies for GDM, with significant improvements in hyperglycemia and insulin resistance in most patients through nonpharmacological interventions. Health education is currently the primary means of correcting patients’ dietary and exercise habits, aiming to improve behavior by increasing knowledge and correcting misconceptions.^10^ Traditional health education, which is often a one-way transmission of information, struggles to engage patients proactively and can even evoke resistance, highlighting its shortcomings.^11^ Previous behavioral research on patients with diabetes has shown that multicomponent interventions are effective in improving glucose control. For example, Hood et al.^12^ conducted a meta-analysis of 15 randomized controlled trials involving 997 youths under 19 years old with type 1 diabetes. The results indicated that, although the overall mean effect size of the comparison between the intervention and control groups was small (0.11, 95% CI: −0.01 to 0.23), interventions targeting emotional, social, or family processes, in addition to direct behavioral processes, were more effective in improving glycemic control. They concluded that multicomponent interventions are more powerful, and future research should focus on refining such interventions. Other studies^13–15^ also support the idea that comprehensive interventions, especially those addressing emotional or social aspects, play a positive role in diabetes management.

The information–motivation–behavioral skills (IMB) model, introduced by Fisher et al. in 1992,^16^ has been widely applied in social and health psychology fields, offering unique advantages over other behavioral theories. The model posits that health-related information, motivation, and behavioral skills are all essential for health behavior change, and that the presence of all elements within the model is necessary for the desired health behavior change to occur. The IMB model has demonstrated significant effects in various populations, including healthy individuals, cardiovascular and respiratory departments, oncology, geriatrics, and special groups such as patients with HIV.^17,18^ However, its application in perinatal management, particularly for GDM behavioral intervention, has been minimal, and further research is needed to explore its potential in this area. This study aims to determine the efficacy of the IMB model-based nursing intervention in enhancing maternal and neonatal outcomes for women with GDM, thereby addressing a critical gap in perinatal care management.

## Methods

### Patient recruitment

This study involved 240 patients diagnosed with GDM who were undergoing regular prenatal care at Aksu First People’s Hospital between June 2023 and May 2024. Participants were randomly assigned to either the IMB group or the traditional health education group, with 120 patients in each group. The randomization process was conducted using a simple random sampling method based on a random number table to ensure equal distribution and minimize selection bias.

#### Inclusion criteria

1. Meeting the diagnostic criteria for GDM as established by the International Association of Diabetes and Pregnancy Study Groups and having undergone a 75-g oral glucose tolerance test between 24 and 28 weeks of gestation using a one-step method.
2. The current pregnancy is a singleton.
3. Willingness to participate in the study, as evidenced by the signing of an informed consent form.

#### Exclusion criteria

1. Individuals with auditory or visual impairments that could affect communication.
2. A history of neurological or psychiatric disorders.
3. A history of previous GDM.
4. Pre-existing diabetes mellitus.
5. Manifest diabetes during pregnancy, defined as a fasting plasma glucose (FPG) ≥7.0 mmol/L (126 mg/dL), or a 2-hour postload blood glucose ≥11.1 mmol/L (200 mg/dL), or a random blood glucose ≥11.1 mmol/L (200 mg/dL).
6. Significant traumatic events during pregnancy, such as the death of a spouse.

**FIG. 1.**
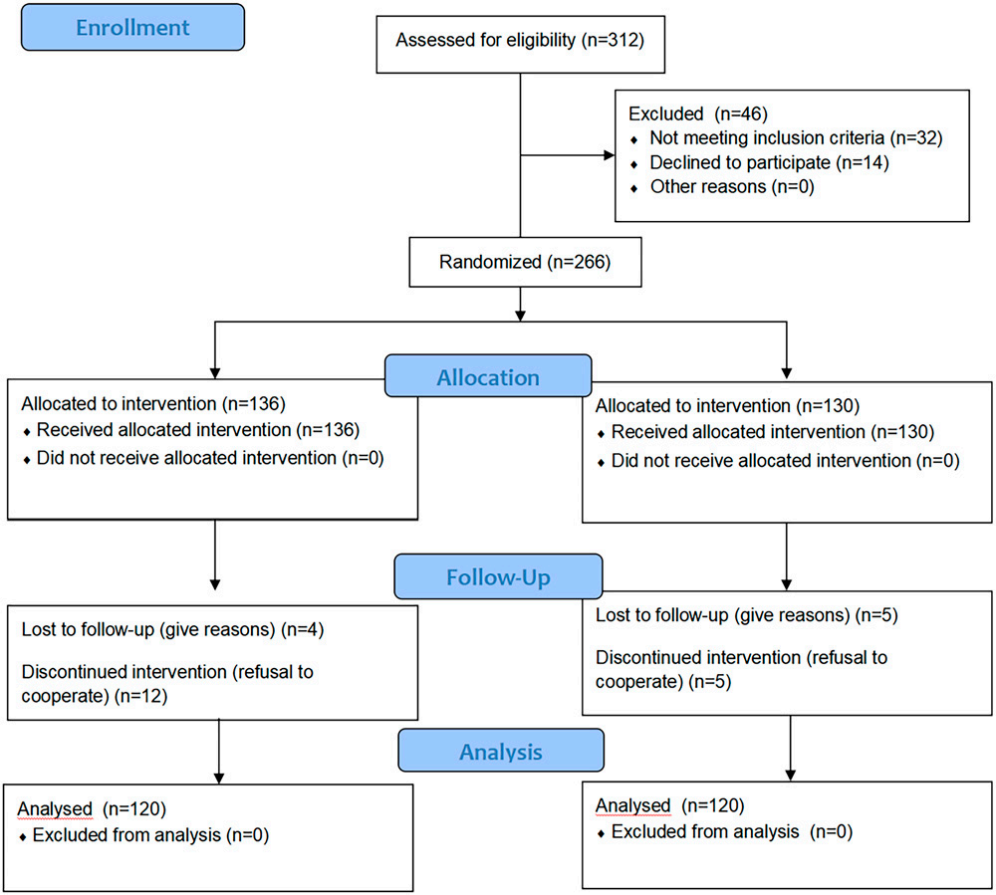
CONSORT flow diagram of this study.

The study was approved by the Medical Ethics Committee of Aksu First People’s Hospital, and all participants provided informed consent in accordance with ethical standards. Information on recruitment for this study is shown in Figure 1.

### Intervention methods

#### IMB group

The IMB group received nursing interventions based on the IMB model. The intervention period lasted for 6 weeks, with one session per week, each lasting 60 minutes, totaling six sessions. The specific intervention steps are as follows:

Establishment of the IMB education team: An educational team was formed, comprising a head nurse, a diabetes specialist nurse, two senior responsibility nurses, and an endocrinologist.

Information intervention: Drawing on the content and format of the “Diabetes Illustrated Dialogue” tool, developed by the International Diabetes Federation and a health interaction company,^19^ health education session was conducted. The content included the concept of GDM, psychological dynamics upon GDM diagnosis, risks of GDM to mothers and infants, effective blood glucose control methods, postpartum precautions and prognosis, support networks, and health management teams for pregnant women with GDM. Interactive health education methods between nurses and patients, as well as among patients, were encouraged to promote active questioning and discussion, thereby deepening the understanding of the risks of GDM and the importance of self-management. Relevant content on the management of gestational diabetes and the adverse effects of hyperglycemia on birth outcomes was transformed into electronic educational materials and made accessible *via* QR codes for patients to study at their convenience.

Motivational intervention: This included face-to-face interviews with patients to encourage them to express their thoughts on their symptoms, guiding them to recognize the importance of self-care in managing gestational diabetes. Peer support was facilitated by inviting mothers with well-controlled blood sugar to share their experiences, providing mutual encouragement and learning. Family support was enhanced through increased communication with family members, encouraging them to supervise and remind patients to adhere to lifestyle changes and regular blood glucose monitoring, thereby increasing psychological support and helping patients face the disease positively.

Behavioral skills intervention: Experiential health education was used to enhance behavioral skills among pregnant women with diabetes. This included experiential blood glucose monitoring (where patients were first taught how to use a glucometer, then guided to self-monitor and record blood glucose, with corrections made for any errors and praise given for correct operations to reinforce memory of proper behavior), dietary experiential education (where a professional dietitian explained dietary components, calorie calculation, and reasonable dietary arrangements for diabetes, followed by patients planning their own meals based on the dietitian’s instructions, which were then assessed and adjusted by the dietitian to give patients a more intuitive understanding of dietary structure), and experiential exercise education (where patients were guided on appropriate exercise methods, such as starting yoga or walking for at least 30 minutes after a meal, without intense exercise, and were taught to self-test blood glucose before and after exercise to intuitively feel the changes in blood glucose). In addition, experiential medication use was included (where patients were first instructed on the principles and precautions of hypoglycemic medication use, step therapy principles, and the timing, site, and method of insulin injection, then guided to independently complete the use of hypoglycemic medication and insulin injection).

To ensure the fidelity of the IMB-based interventions, the study team regularly reviewed session records, which detailed the topics covered, the duration of each activity, and the level of patient participation. The team also directly observed at least one intervention session per patient group to assess whether the interventions were delivered as planned. After each observation, the team provided constructive feedback to the intervention providers, focusing on areas such as maintaining patient engagement during information sharing, effectively facilitating peer support, and accurately demonstrating behavioral skills.

#### Traditional health education group

The traditional health education group received health education that included knowledge about GDM, blood glucose monitoring methods, and proper exercise. The content was the same as that provided to the IMB group, but the teaching method differed, employing a health lecture format and traditional teaching methods, which mainly involved one-way knowledge transfer to provide patients with scientific guidance on diet and exercise. The intervention period was also 6 weeks, with one session per week, each lasting 60 minutes, totaling six sessions.

The study team monitored the fidelity of the traditional health education by checking lecture content consistency, observing teaching sessions, and collecting feedback from patients on the delivery of the education.

#### Observational indicators

The study assessed the following parameters to evaluate the effectiveness of the interventions:

##### General demographic information

Custom-designed questionnaires were used to collect data on maternal age, gestational age, and prepregnancy body mass index.

##### Insulin treatment during intervention

The number of participants who required insulin therapy due to inadequate blood glucose control after 1–2 weeks of dietary and exercise intervention was recorded.

##### Fasting Plasma Glucose

Blood samples (3–4 mL) were collected from the fasting participants in the morning before the intervention and after 6 weeks of intervention. FPG levels were measured using the glucose oxidase method (Beckman Coulter, Inc., USA).

##### Glycosylated hemoglobin

Glycosylated hemoglobin (HbA1c) levels were assessed before and 6 weeks after the intervention to evaluate long-term glycemic control. The measurements were performed using an electrochemiluminescence assay (Roche Diagnostics, Germany).

##### Adverse maternal and infant delivery outcomes

These outcomes were recorded and included events such as preterm birth, cesarean section, macrosomia, and neonatal complications.

##### Postpartum glucose metabolism abnormalities

The incidence of diabetes, impaired glucose tolerance, and impaired fasting glucose was assessed 12 weeks after delivery.

### Statistical methods

Data analysis was conducted using SPSS 25.0 software (International Business Machines Corporation [IBM], Armonk, New York, US). Quantitative data that were normally distributed are presented as the mean and standard deviation. Intergroup comparisons for these data were performed using the independent samples *t* test, while intragroup comparisons were conducted using the paired samples *t* test. Categorical data are expressed as frequencies (percentages), and comparisons were made using the chi-square (χ^2^) test or Fisher’s exact probability method, as appropriate. A *p*-value < 0.05 was considered to indicate statistical significance.

Prior to the study, a power analysis was conducted using G*Power software. Based on an expected medium effect size (Cohen’s d = 0.5), a significance level of *p* < 0.05, and a desired power of 80%, the required sample size was calculated to be 104 participants. This sample size (240 participants) was determined to be sufficient to detect significant differences.

## Results

### Baseline characteristics

The baseline characteristics of the pregnant women with GDM in both the IMB group and the control group are presented in Table 1. The comparison of general demographic and clinical information between the two groups showed no significant differences (*p >* 0.05), indicating that the randomization process was effective in creating comparable groups at the start of the study.

**Table 1.** Comparison of General Information of Pregnant Women with Gestational Diabetes Mmellitus in Both Groups

Item	IMB group (*n* = 120)	Traditional health education group (*n* = 120)	*t*/χ^2^	*p*
Gestational age (weeks, x ± s)	27.6 ± 0.9	25.9 ± 0.7	1.8	0.074
Age (years, x ± s)	31.7 ± 4.6	32.2 ± 5.2	−1.4	0.161
Prepregnancy BMI (kg/m², x ± s)	23.9 ± 3.8	24.1 ± 3.7	−0.5	0.618
Parity (No. of women)			0.2	0.646
Nulliparous	65 (54.17%)	63 (52.50%)		
Multiparous	55 (45.83%)	57 (47.50%)		
Education level (No. of women)			0.1	0.752
Junior high school or below	12 (10.00%)	11 (9.17%)		
Senior high school or technical secondary school	33 (27.50%)	31 (25.83%)		
Junior college	38 (31.67%)	39 (32.50%)		
Bachelor’s degree or above	37 (30.83%)	39 (32.50%)		
Family history (number of women)	22 (18.33%)	26 (21.67%)	1.2	0.274

BMI, body mass index.

### Comparison of insulin treatment during the intervention period

In this study, there were no instances of noncompliance, loss to follow-up, or withdrawal from the study in either group. The participants included were those who regularly attended prenatal check-ups at our hospital, demonstrating high levels of compliance. During the intervention period, three participants in the IMB group required insulin therapy, compared to 16 participants in the traditional health education group. The rate of insulin treatment during the intervention was significantly lower in the IMB group than in the traditional health education group (χ^2^ = 4.745, *p* = 0.003).

### Comparison of blood glucose control between the two groups before and after intervention

The comparison of blood glucose control between the IMB group and the traditional health education group before and after the intervention is presented in Table 2. Both groups demonstrated a significant reduction in FPG and HbA1c levels after the intervention, indicating effective blood glucose control.

**Table 2.** Comparison of Blood Glucose Control Between the Two Groups Before and After Intervention

Group	Number of cases	FPG (mmol/L)	FPG (mmol/L)	HbA1c (%)	HbA1c (%)
		Before intervention	After intervention	Before intervention	After intervention
IMB group	120	8.05 ± 0.97	4.95 ± 0.86^*^	6.60 ± 0.97	4.98 ± 0.88^*^
Traditional health education group	120	7.98 ± 1.06	6.00 ± 0.88	6.45 ± 0.92	5.78 ± 0.90^*^
*t* value		0.55	7.40	1.20	5.30
*p*-value		0.59	<0.001	0.24	<0.001

^*^ Statistically significant difference compared to before intervention within the same group, with *p-*values < 0.05.

FPG, fasting plasma glucose; HbA1c, glycosylated hemoglobin; IMB, Information-Motivation-Behavioral Skills.

### Comparison of maternal adverse delivery outcomes between the two groups

The comparison of maternal adverse delivery outcomes between the IMB group and the traditional health education group is detailed in Table 3. The data reveal significant differences in the rates of certain complications, suggesting the impact of the IMB model-based intervention on maternal outcomes (*p* < 0.05).

**Table 3.** Comparison of Maternal Adverse Delivery Outcomes Between the Two Groups

Group	No. of cases	Preeclampsia	Preterm birth	Cesarean section	Polyhydramnios	Postpartum hemorrhage	Postpartum infection
IMB group	120	1 (0.83%)	5 (4.17%)	18 (15.00%)	4 (3.33%)	6 (5.00%)	1 (0.83%)
Traditional health education group	120	6 (5.00%)	7 (5.83%)	48 (40.00%)	10 (8.33%)	12 (10.00%)	4 (3.33%)
χ^2^ value			0.214	21.844	3.741	2.136	
*p*-value		0.654^*^	0.651^#^	<0.001	0.083	0.150	0.383

^*^ Statistical significance using Fisher’s exact test.

^#^ Statistical significance using Cochran’s Q test.

### Comparison of neonatal adverse outcomes between the two groups

The comparison of neonatal adverse outcomes between the IMB group and the traditional health education group is presented in Table 4. The data indicate a significant difference in the incidence of certain neonatal complications between the two groups, suggesting a potential impact of the IMB model-based intervention on neonatal health outcomes (*p* < 0.05).

**Table 4.** Comparison of Neonatal Adverse Outcomes Between the Two Groups

Group	No. of cases	Macrosomia	Neonatal respiratory distress syndrome	Jaundice	Hypocalcemia	Hypoglycemia
IMB group	120	6 (5.00%)	2 (1.67%)	18 (15.00%)	2 (1.67%)	9 (7.50%)
Traditional health education group	120	10 (8.33%)	12 (10.00%)	30 (25.00%)	4 (3.33%)	15 (12.50%)
χ^2^ value		1.750	7.201	3.014	0.336	2.547
*p*-value		0.185	0.007	0.083	0.584^#^	0.113

^#^ Statistical significance using Cochran’s Q test.

### Comparison of occurrence of abnormal postpartum glucose metabolism between the two groups

The incidence of abnormal postpartum glucose metabolism was compared between the IMB group and the traditional health education group, with the results presented in Table 5. The data indicate a significant difference in the rates of postpartum glucose metabolism abnormalities between the two groups, suggesting the potential effectiveness of the IMB model-based intervention in reducing the risk of such conditions (*p* < 0.05).

**Table 5.** Comparison of Occurrence of Abnormal Postpartum Glucose Metabolism Between the Two Groups

Group	No. of cases	Diabetes	Impaired glucose tolerance	Impaired fasting glucose	Total
IMB group	120	1 (0.83%)	6 (5.00%)	8 (6.67%)	15 (12.50%)
Traditional health education group	120	4 (3.33%)	12 (10.00%)	14 (11.67%)	30 (25.00%)
χ^2^ value			4.025	3.210	10.896
*p*-value		0.245^*^	0.045	0.073	<0.001

^*^ Statistical significance using Fisher’s exact test.

## Discussion

The IMB model posits that information and motivation directly influence health behaviors, primarily by affecting behavioral skills, which in turn can also directly shape health behaviors. This model has been effectively utilized to achieve behavior change and enhance patient proactivity and compliance.^20^ Initially applied to manage behaviors in patients with HIV/AIDS, the IMB model has been successful in elevating cognitive levels and correcting erroneous behaviors, demonstrating positive outcomes in both domestic and international health education and behavioral interventions for HIV/AIDS.^21,22^ In the context of the IMB model, information serves as the foundation for behavior change, emphasizing the importance of disease-related knowledge as a prerequisite for adopting preventive measures, which subsequently impacts health behavior transformation.^23^ Studies have shown a positive correlation between changes in health behavior and the level of disease knowledge.^24,25^

Ranahan et al.^26^ applied the IMB model to self-behavior management interventions for kidney transplant patients, resulting in improved cognitive levels and better self-behavior management. Similarly, Lee et al.^27^ found that health education based on the IMB model could enhance the self-management capabilities of patients with respiratory tract infections, leading to better infection control. These findings suggest that health education and behavioral interventions grounded in the IMB model can play a significant role in the management of various diseases.

Our study’s results indicate that both FPG and HbA1c levels decreased after the intervention in both groups, but the IMB group showed lower levels compared to the traditional health education group. In addition, the rate of insulin treatment during the intervention period was lower in the IMB group, suggesting that the IMB model-based nursing intervention, through interactive health education and experiential behavioral skill interventions, can more effectively manage patients’ dietary and exercise behaviors. The correction of these health behaviors is beneficial for improving blood sugar control, consistent with the findings of Lin et al.^28^

Furthermore, when patients with GDM have poor blood sugar control, the high blood sugar in the mother can also raise the fetal blood sugar, leading to increased fetal insulin secretion. This excess of blood sugar and insulin can result in increased fat and protein synthesis in the fetus, often manifesting as macrosomia, which is characterized by an enlarged upper body. This condition can lead to adverse outcomes such as fetal distress and preterm birth, increasing the risk of difficult labor and the rate of cesarean sections.^29,30^ In our study, the IMB group had lower rates of cesarean sections and neonatal respiratory distress syndrome, indicating that the IMB model-based nursing intervention improved patient health behaviors, controlled blood sugar levels promptly, reduced the impact of blood sugar on both mother and fetus, and improved maternal and infant outcomes. According to literature reports,^31,32^ if patients with GDM do not maintain a reasonable diet and increase their body weight after childbirth, it can lead to the continuation of abnormal glucose metabolism. Lv et al.^33^ found that the incidence of postpartum diabetes in 210 patients with GDM was 10.48%. In this study, only one case of diabetes occurred in the IMB group within 12 weeks after delivery (incidence rate 0.83%), while the incidence rate in the traditional health education group was 3.75%, which was slightly lower than the above report, which may be related to the different follow-up durations. The short follow-up time in our hospital did not allow for an assessment of long-term postpartum diabetes development, which is a limitation of this study. However, the total incidence of abnormal glucose metabolism in the IMB group was lower than in the traditional health education group, suggesting that the IMB model-based nursing intervention provided adequate health education during pregnancy, enabling patients to actively correct their diet and exercise behaviors. These healthy behaviors were sustained postpartum, allowing the patients to control their diet and exercise reasonably, thus avoiding the continuation of diabetes, which is consistent with current research findings.^34^

However, due to the small sample size of this study, the results may be biased,^21^ and the conclusions should be considered with caution, pending further validation through large-sample, long-term follow-up studies.

In summary, the nursing intervention based on the IMB model can correct the dietary and behavioral habits of patients with GDM, improve glucose metabolism, reduce adverse maternal and infant outcomes, and effectively prevent the continuation of abnormal glucose metabolism postpartum.

## Data Availability

The experimental data used to support the findings of this study are available from the corresponding author upon request.

## Abbreviations Used

FPG: fasting plasma glucose
GDM: gestational diabetes mellitus
IMB: information–motivation–behavioral skills
